# Multiple Endocrine Neoplasia Type 1 Regulates TGFβ-Mediated Suppression of Tumor Formation and Metastasis in Melanoma

**DOI:** 10.3390/cells13110973

**Published:** 2024-06-04

**Authors:** Julien Boudreault, Lucie Canaff, Mostafa Ghozlan, Ni Wang, Vito Guarnieri, Antonio Stefano Salcuni, Alfredo Scillitani, David Goltzman, Suhad Ali, Jean-Jacques Lebrun

**Affiliations:** 1 Cancer Research Program, Department of Medicine, Research Institute of McGill University Health Center, Montreal, QC H4A 3J1, Canada; julien.boudreault2@mail.mcgill.ca (J.B.); lucie.canaff@gmail.com (L.C.); mostafa.ghozlan@mcgill.ca (M.G.); ni.wang79@gmail.com (N.W.); david.goltzman@mcgill.ca (D.G.); suhad.ali@mcgill.ca (S.A.); 2 Division of Medical Genetics, Fondazione IRCCS Casa Sollievo della Sofferenza, 71013 San Giovanni Rotondo, Italy; vitoguarnieri@gmail.com; 3 Endocrinology and Metabolism Unit, University-Hospital S. Maria della Misericordia, 33100 Udine, Italy; salcuni.antonio@libero.it; 4 Endocrinology Unit, Fondazione IRCCS Casa Sollievo della Sofferenza, 71013 San Giovanni Rotondo, Italy; alfredo.scillitani@gmail.com

**Keywords:** melanoma, TGFβ signaling, *MEN1*, tumor suppression, metastasis, *MEN1* missense and frameshift mutations, proteasome inhibitor, CHIP RNAi targeting

## Abstract

Over the past few decades, the worldwide incidence of cutaneous melanoma, a malignant neoplasm arising from melanocytes, has been increasing markedly, leading to the highest rate of skin cancer-related deaths. While localized tumors are easily removed by excision surgery, late-stage metastatic melanomas are refractory to treatment and exhibit a poor prognosis. Consequently, unraveling the molecular mechanisms underlying melanoma tumorigenesis and metastasis is crucial for developing novel targeted therapies. We found that the multiple endocrine neoplasia type 1 (MEN1) gene product Menin is required for the transforming growth factor beta (TGFβ) signaling pathway to induce cell growth arrest and apoptosis in vitro and prevent tumorigenesis in vivo in preclinical xenograft models of melanoma. We further identified point mutations in two MEN1 family members affected by melanoma that led to proteasomal degradation of the MEN1 gene product and to a loss of TGFβ signaling. Interestingly, blocking the proteasome degradation pathway using an FDA-approved drug and RNAi targeting could efficiently restore MEN1 expression and TGFβ transcriptional responses. Together, these results provide new potential therapeutic strategies and patient stratification for the treatment of cutaneous melanoma.

## 1. Introduction

Cutaneous melanoma is a deadly and aggressive cancer accounting for approximately 80% of skin cancer-related deaths [1]. Globally, melanoma is the fifteenth most common cancer, with 230,000 diagnosed cases per year and 55,000 deaths. Furthermore, the incidence of melanoma has significantly increased during the past fifty years [2]. Melanoma is one of the most prevalent cancers among younger adults aged >20–35 years worldwide [3]. Based on histopathology and prognostic outcomes, melanomas are conventionally classified into four clinical stages. While patients with stage I melanoma have localized primary tumors that can be removed by surgical excision [4], stage IV patients exhibit secondary metastatic tumors to the lung, liver, bones, or brain and are refractory to traditional chemotherapy [5]. As a result, while 5-year and 15-year survival rates are very good in stage I patients (97% and 85%, respectively), they plummet to only 15% and 5%, respectively, in patients with stage IV melanoma [1,2]. In addition to UV radiation, the primary environmental factor predisposing patients to cutaneous melanoma and other genetic and molecular factors involved in the genesis of this disease have yet to be fully characterized [6]. Hence, understanding the molecular and signaling mechanisms leading to melanoma development and progression is essential for developing better targeted treatments.

Melanoma tumorigenesis results from mutations in genes implicated in the regulation of various biological processes, including cell growth and proliferation (BRAF, NRAS, NF1, PTEN, and KIT), apoptosis (TP53), and cell immortalization (hTERT) [7,8]. While mutations in the mitogenic RAS-RAF-MEK-ERK signaling pathway are very frequent, other signaling pathways, such as the Jnk/c-Jun, Wnt, NF-κB, PI3K/AKT, JAK/STAT, and TGFβ pathways, have also been implicated in the tumorigenesis process [9,10,11,12]. The TGFβ signaling pathway plays an essential role in both normal melanocytes and melanoma cells. TGFβ signals through a complex of two serine/threonine kinase receptors and intracellular Smad proteins (Smad2, 3, and 4). In melanoma, the TGFβ/Smad3 signaling pathway strongly suppresses tumorigenesis by blocking cell growth, immortalization, and cancer stem cell self-renewal activities, and by inducing cell death and autophagy [13,14,15].

Multiple endocrine neoplasia type 1 (MEN1) is an autosomal dominant disorder affecting the endocrine system and is characterized by the concomitant occurrence of tumors in the pancreas as well as in the parathyroid and anterior pituitary glands. The *MEN1* gene encodes Menin, a 610-amino-acid protein that interacts with numerous protein partners, including several transcription factors [16]. Menin plays a significant role in cell-cycle regulation by inducing cyclin-dependent kinase inhibitor (*CDKI*) gene expression [17,18]. Interestingly, Menin was found to leverage TGFβ signaling at the transcriptional level, thus facilitating its cytostatic and differentiation functions [19,20].

Notably, non-endocrine tumors have also been reported in *MEN1* patients. These include skin tumors of mesenchymal origin, such as angiofibromas, collagenomas, lipomas, and malignant melanomas [21,22,23]. Loss of heterozygosity (LOH) of chromosome 11q13 (the *MEN1* gene locus) was detected in six melanoma tumors, and deletion of the *MEN1* locus was found in 19 patients with sporadic metastatic melanoma. Another study suggested that multiple melanoma tumor suppressors are localized on chromosome 11q, which includes the *MEN1* region [24]. These observations therefore raise the possibility of a causal association between *MEN1* and melanoma. It was shown that Menin suppresses malignant phenotypes of melanoma through its involvement in PTN signaling [25]. Interestingly, a paradoxical role of *MEN1* was discovered in breast cancer patients, as its high expression is correlated with poorer overall survival [26].

In this study, we identified the TGFβ/Smad3/*MEN1* signaling axis as a potent tumor-suppressor pathway in cutaneous melanoma. Moreover, genetic analysis of two *MEN1* family members affected by melanoma revealed the presence of specific point mutations within the *MEN1* gene. We found that these point mutations induce *MEN1* gene product degradation, leading to further loss of TGFβ signaling. Moreover, we showed that by targeting the co-chaperone of the proteasome degradation pathway, CHIP could restore Menin expression and TGFβ signaling in these melanoma cells. Overall, this study defines the TGFβ/*MEN1* axis as a potent tumor-suppressor pathway in cutaneous melanoma and provides novel perspectives for tailor-made targeted therapies for this highly lethal malignancy.

## 2. Materials and Methods

### 2.1. Reagents

Recombinant human TGFβ (PeproTech, Saint-Laurent, QC, Canada), RPMI 1640 tissue culture medium, DMEM (HyClone Logan, South Logan, UT, USA), fetal bovine serum and penicillin/streptomycin (Gibco, Waltham, MA, USA), branched polyethyleneimine (Sigma-Aldrich, Oakville, ON, Canada), Moloney murine leukemia virus (MMLV) reverse transcriptase, and random primers (Life Science, N. St. Petersburg, FL, USA) were used. The sequence of the control siRNA was sc-37007, and that of the CHIP siRNA was sc-43555 (Santa Cruz, Santa Cruz, CA, USA). Missense mutations were generated with the Quik Change Site-Directed Mutagenesis Kit from Stratagene (La Jolla, CA, USA).

### 2.2. Antibodies

β-Tubulin (3F32G) (Santa Cruz); anti-Flag M2 monoclonal antibody (Sigma); MEN1 (2605) (Abcam, Cambridge, UK); P21 (C-19) (Santa Cruz, Cat#sc-397); SMAD2-3 (Santa Cruz, Cat#sc-6032); SMAD4 (Santa Cruz, Cat#sc-7966); Caspase-3 (H-277) (Santa Cruz, Cat#sc-7148); and c-myc (9E10) (Santa Cruz, Cat#sc-40)

### 2.3. Cell Lines

The cells were cultured at 37 °C and 5% CO_2_ in RPMI 1640 (BLM, WM793B, WM278, WM1232, DAUV) or DMEM (HEK293, SKMEL28, and a375m) supplemented with 10% FBS and 1% penicillin/streptomycin. Additional information is provided in Table 1.

### 2.4. TGFβ Treatment

Cell monolayers were grown in complete medium to 60% confluence, starved overnight in serum-free medium (0% FBS), and treated with a final concentration of 200 pM human recombinant TGFβ1 for the indicated time periods.

### 2.5. Quantitative Real-Time PCR

Total RNA was extracted using Trizol ^TM^ (Invitrogen, Burlington, ON, Canada). RNA was reverse transcribed using M-MLV reverse transcriptase and random primers (Invitrogen) according to the manufacturer’s protocol. cDNA amplification was performed via quantitative real-time PCR (qPCR) with SsoFast™ EvaGreen^®^ Supermix (Bio-Rad, Mississauga, ON, Canada) using a Rotor-Gene™ 6000 Real-time Analyzer (Corbett Life Sciences, Mortlake, NSW, Australia). Human GAPDH was used as a housekeeping gene. The sequences of primers used are listed in the Table 2:

### 2.6. Clonogenic Assay

Melanoma cells (WM278) were grown in a 6-well plate (1000 cells) in complete RPMI medium (10% FBS). The medium was replenished after 1 week, after which the cells were fixed, stained (0.5% *w*/*v* crystal violet, 20% *v*/*v* methanol) at the endpoint and washed with PBS.

### 2.7. Flow Cytometry

For cell-cycle analysis, cells were stimulated with or without TGF-β (200 pM) for 24 h or 48 h in 1% FBS. The cells were washed and resuspended in PBS at 1 × 10^6^ cells/mL and fixed in ice-cold water by dropwise addition of 70% ethanol while vortexing. The mixture was incubated on ice for 30 min after fixation. For analysis, the cells were resuspended in a mixture of 50 μg/mL propidium iodide, 50 μg/mL RNase A, 10 mM HEPES (pH 7.4), 2.5 mM CaCl_2_ and 140 mM NaCl. The mixture was incubated for 15 min at room temperature.

For the apoptosis assay, cells were stimulated with or without TGF-β (200 pM) for 48 h in full medium. The Annexin V Kit (Santa Cruz), using an FITC-conjugated antibody and PI staining, was used according to the commercial protocol procedure.

Both cell-cycle distribution and apoptotic/pre-apoptotic/live cells were analyzed using a BD FACSCanto flow cytometer with a FACSDiva (BD Biosciences, Mississauga, ON, Canada) and FlowJo V10 software (FlowJo, LLC, Ashland, OR, USA).

### 2.8. Immunoblotting

Cells were lysed at 4 °C for 15 min in RIPA buffer (1 mM DTT, 1 mM EDTA, 1 mM EGTA, 150 mM NaCl, 50 mM Tris-HCl, pH 7.41% Triton X-100) supplemented with protease inhibitors (10 μg/mL aprotinin and leupeptin, 2 μg/mL pepstatin A, and 1 mM PMSF). Total lysates were immunoblotted via SDS–PAGE using specific antibodies. Immunoreactivity was visualized by chemiluminescence using Clarity™ Western ECL Substrate and detected using a ChemiDoc™ Imaging System. Densitometric analysis of protein levels was performed using Image Lab™ Software version 6.0.1 (Bio-Rad, Mississauga, ON, Canada).

### 2.9. Lentiviral Generation and Infection

HEK293T cells were cultured in T75 flasks to 90% confluence in complete medium and transfected with scrambled or *MEN1* shRNA or the packaging plasmid pMD2. G and psPAX2 using Opti-MEM^®^ (Invitrogen) and branched polyethyleneimine (Sigma). Melanoma cells were cultured in cell culture medium supplemented with lentivirus particles and cultured in 6-well plates until they reached 70–80% confluence. Afterwards, the cells were infected with 100 μL of lentivirus in the presence of hexadimethrine bromide and polybrene (8 μg/mL). Cells were selected with 1 μg/mL puromycin for 3 days post infection.

### 2.10. Generation of MEN1 CRISPR Knockout Cells

Guide RNAs (gRNAs), a nontargeting control (SCR, scramble), or a sequence targeting *MEN1* or *Smad2/3/4* (Table 3) were cloned and inserted into a lentiCRISPRv2 plasmid for lentiviral packaging [27]. Melanoma cells were grown in 6-well plates to 50% confluence in antibiotic-free medium and infected with 100 μL of lentivirus. For the a375m and DAUV cell lines, the cells were incubated overnight, and the medium was replenished the next day with fresh complete medium for 2 days. For BLM, WM1232, and WM278, cells were infected by spinfection (2 h, 1500 G and 33 °C), the medium was replenished immediately after centrifugation, and the cells were allowed to grow for 2 days. The pool of resistant cells that formed stable CRISPR-knockout cells was expanded in complete medium (supplemented with 10% FBS) and selected with 0.5 μg/mL (DAUV) or 1 μg/mL (a375m, BLM, WM1232 and WM278) puromycin. Before proceeding with the experiments, the knockout efficiency was verified using Western blotting.

### 2.11. Luciferase Assay

DAUV cells were transfected with 1.5 μg of the promoter luciferase reporter construct, 1.5 μg of the β-galactosidase (pCMV-lacZ) expression vector and 9 μg of polyethyleneimine (PEI) 25,000. The cells were serum-starved in RPMI overnight and cultured with or without TGFβ (200 pM) for 24 h. The cells were washed in PBS and lysed in 100 μL of passive lysis buffer (25 mM glycylglycine, 15 mM MgSO_4_, 4 mM EGTA, 1 mM DTT and 1% Triton X-100) on ice. The supernatants were collected by centrifugation (14,000 rpm, 10 min, 4 °C). Forty-five microliters of the clear cell lysates was mixed with 5 μL of cocktail buffer (30 mM ATP, 100 mM KH_2_PO_4_ pH 7.8, 100 mM MgCl_2_), and the luciferase activity was measured after the injection of 50 μL of 0.25 mM D-luciferin using a luminometer, where the luminescence levels were expressed as relative light units (RLUs). In parallel, 5 μL of lysate was mixed with 45 μL of ONPG (6 mg/mL) in β-Gal buffer (60 mM Na_2_HPO_4_, 40 mM NaH_2_PO_4_, 50 mM βME, 10 mM KCl, 1 mM MgCl_2_) and incubated at 37 °C for 1 h. The OD was measured at 420 nm, and the normalized luciferase activity of each lysate was calculated by dividing the RLU value of the luciferase activity by the corresponding β-galactosidase activity of the co-transfected β-gal vector.

### 2.12. Subcutaneous Tumor Xenografts

Male NSG mice were bred from mouse breeding pairs that were purchased from The Jackson Laboratory and were used for the experiments at the age of 7 weeks. The mice were housed and handled in accordance with approved guidelines of the Canadian Council on Animal Care (CCAC) under the conditions and procedures approved by the Animal Care Committee of McGill University (AUP # 7497).

For tumor xenografting, the mice were randomized into two groups that received 1 × 10^6^ MEN1 or scrambled knockout stable cells (BLM, WM1232 and WM278) per mouse via the subcutaneous route. Tumor volumes were calculated according to the formula below, and tumor growth curves were generated.

$$\frac{4}{3}*\pi *\left(\frac{Length}{2}\right)*{\left(\frac{Width}{2}\right)}^{2}$$

Sequence analysis of the *MEN1* gene of Leukocyte DNA was performed via standard methods. Exons 2–10 of the MEN1 gene were amplified as described previously [28]. The gel-purified PCR products were directly sequenced.

### 2.13. Statistics

Data were collected from three or more independent experiments and are expressed as arithmetic means. All error bars are standard errors of the means (SEMs). Statistical analysis was performed using Student’s t test or one-way ANOVA to compare TGFβ-treated cells to nontreated controls (* *p* < 0.05, ** *p* < 0.01, *** *p* < 0.001).

## 3. Results

### 3.1. TGFβ Induces MEN1 Gene Expression in Melanoma Cells through Smad3

**Figure 1:**
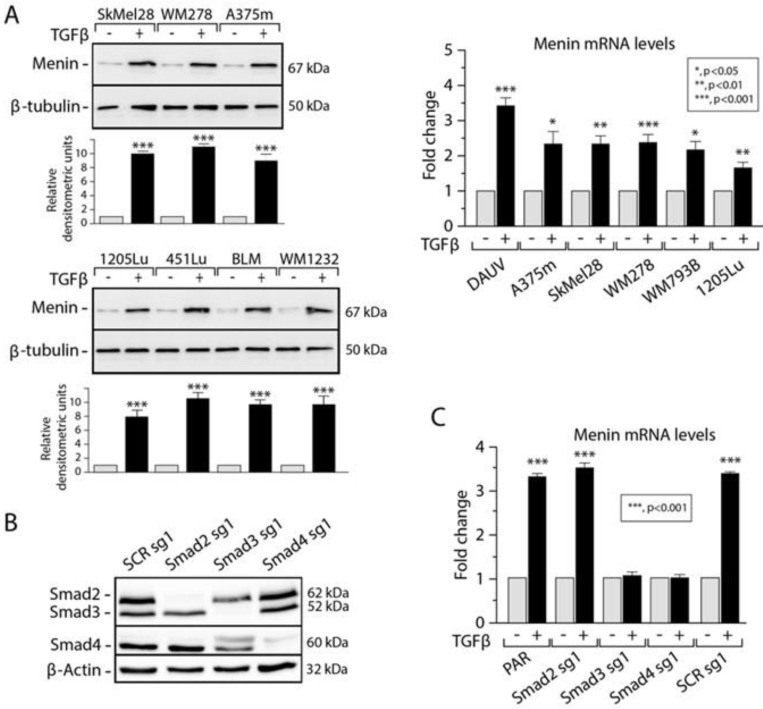
TGFβ induces MEN1 gene expression in melanoma cells through Smad3. (**A**) Regulation of the Menin protein (left panel) and mRNA (right panel) in human melanoma cell lines. Changes in the Menin protein and mRNA expression following TGFβ treatment. Menin and β-tubulin protein expressions were determined via Western blotting. MEN1 mRNA levels were determined by qPCR with *GAPDH* serving as a reference gene. (**B**) Generation of CRISPR/Cas9 *SMAD2/3/4* and control (SCR, scrambled) knockout (KO) DAUV melanoma cell lines. The efficiency of the knockout was assessed using Western blotting. (**C**) MEN1 mRNA expression levels in DAUV CRISPR/Cas9 *SMAD2/3/4* and control (SCR, scrambled) KO cells. MEN1 mRNA levels were determined by qPCR with *GAPDH* serving as a reference gene. *, *p* < 0.05. **, *p* < 0.01. ***, *p* < 0.001.

*MEN1* patients can develop malignancies other than classical endocrine tumors, including skin tumors [29]. Previous work from our laboratory and others showed that the TGFβ signaling pathway acts as a potent tumor suppressor in melanoma [12,13,14,15]. Thus, we investigated whether *MEN1* could relay some of the TGFβ tumor-suppressive response in melanoma. For this purpose, we first investigated whether TGFβ could regulate *MEN1* gene expression in melanoma. A panel of human cutaneous melanoma cell lines with various pathological backgrounds was stimulated with or without TGFβ before assessing Menin mRNA and protein levels by qPCR and Western blot, respectively. As shown in Figure 1A, TGFβ significantly upregulated *MEN1* at both protein (left panel) and mRNA (right panel) levels in all melanoma cell lines.

To determine whether the Smad pathway was involved in mediating the effects of TGFβ on *MEN1* gene expression, we next silenced Smad2, 3, and 4 expressions in the DAUV cell line using CRISPR/Cas9 technology. Specific guide RNAs (gRNAs) targeting these genes were selected, and the efficiency of the Smad2/3/4 (Figure 1B) CRISPR-knockout (KO) efficacy was verified via Western blotting. Interestingly, knocking out Smad3 and Smad4 but not Smad2 impaired TGFβ-mediated Menin expression (Figure 1C), indicating that TGFβ-induced *MEN1* gene expression is Smad3/4-specific and Smad2-independent. Taken together, these results highlight *MEN1* as a novel TGFβ/Smad target in melanoma, further suggesting that *MEN1* may act downstream of TGFβ in melanoma cells.

### 3.2. MEN1 Is Essential for Inhibiting Melanoma Cell Growth and Tumorigenesis

**Figure 2:**
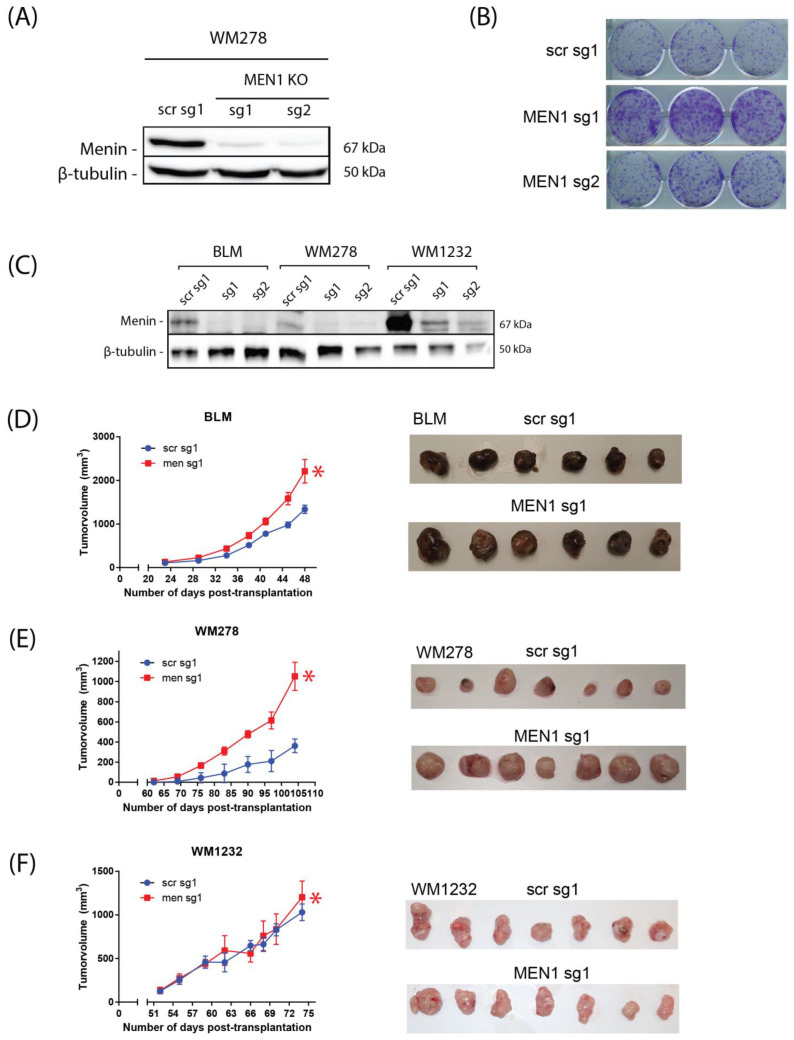
The TGFβ/Smad3/*MEN1* axis is essential for inhibiting melanoma tumor formation in vivo. (**A**) Generation of WM278 melanoma *MEN1* KO and control (SCR, scrambled) CRISPR/Cas9 knockout (KO) cell lines. The KO efficiency of *MEN1* was measured by Western blotting. (**B**) Growth of WM278 cells cultured for 2 weeks and seeded at a low density (1000 cells/well). (**C**) Generation of BLM, WM278, and WM1232 melanoma CRISPR/Cas9 *MEN1* KO. KO efficiency was measured by Western blotting. (**D**–**F**) NSG mice were injected subcutaneously with BLM (**D**), WM278 (**E**), WM1232 (**F**), or scrambled (SCR) or with MEN1 knockout melanoma cells at 1 × 10^6^ cells/mouse. As shown in (**D**–**F**), the left panel shows the mean tumor volumes, and the right panel shows representative images of tumors at the tumor collection endpoint. *, *p* < 0.05 (in red).

The TGFβ/Smad signaling pathway exerts potent tumor-suppressive effects on melanoma [15]. We thus investigated the role and contribution of *MEN1* downstream of TGFβ-mediated growth inhibition. To this end, we generated *MEN1* CRISPR-KOs in WM278 melanoma cells (Figure 2A) and assessed their effects on colony formation assay in vitro. As shown in Figure 2B, we found that silencing *MEN1* strongly increased cell clonogenicity. These results indicate that *MEN1* could have tumor-suppressive effects on melanoma. We next investigated whether the tumor-suppressive effects of the *MEN1* pathway could lead to the inhibition of tumor formation in vivo using preclinical models of melanoma. For this purpose, we generated *MEN1* knockout models in three different melanoma cell lines (WM1232, BLM and WM278). The KO efficacy in all cell lines was verified by Western blotting (Figure 2C). Subsequently, *MEN1* KOs and scr controls were transplanted subcutaneously into immunocompromised NOD/SCID/IL2Rγ-/- (NSG) mice as previously described [15]. Interestingly, as shown in Figure 2D–F, the results showed that mice injected with *MEN1* KO melanoma cells harbored significantly larger tumors than did the control animals. These results are consistent with our aforementioned in vitro data, showing that *MEN1* is required for tumor growth inhibition. The significant increases in primary tumor formation observed in vivo upon *MEN1* silencing further emphasize the central role of *MEN1* in mediating tumor suppression in melanoma.

### 3.3. The TGFβ/Smad3/MEN1 Axis Is Essential for Inducing Cell-Cycle Arrest and Apoptosis in Human Melanoma Cells

**Figure 3:**
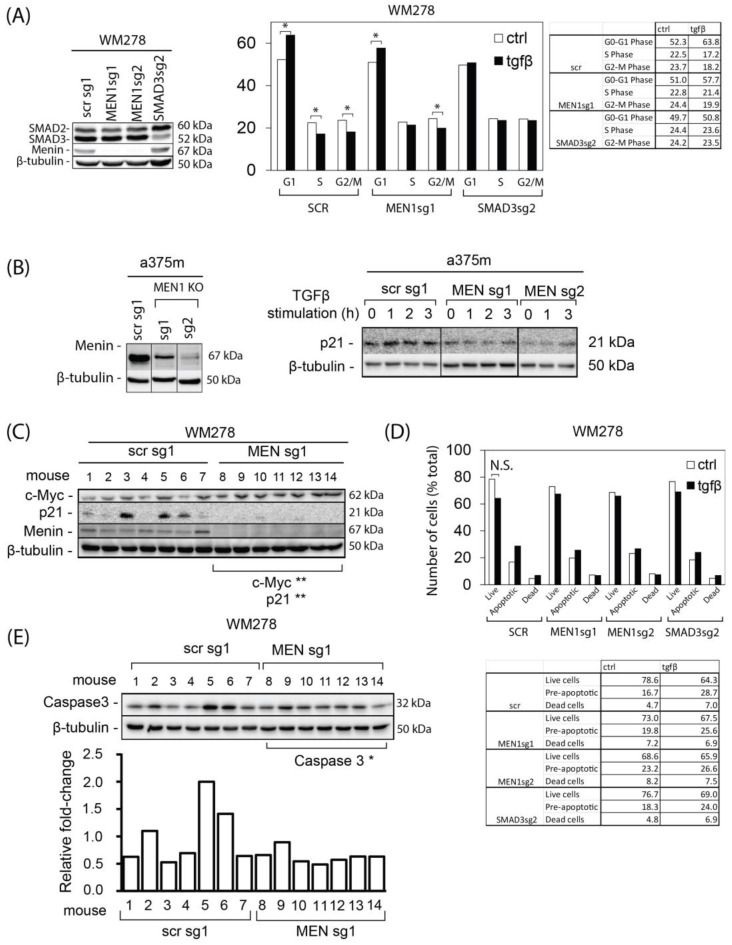
The TGFβ/Smad3/MEN1 axis is essential for inducing cell-cycle arrest in human melanoma cells. (**A**). (left panel) Cell-cycle distribution assessed by flow cytometry analysis of propidium iodide-stained WM278 scrambled, *MEN1* KO, and SMAD3 KO cells following TGFβ treatment (24 h). (right panel) Quantification table displaying the percentage of G1, S, and G2/M phase cells. Cell-cycle experiments were performed in three biological replicates. A two-sample equal variance T-test was used to compare the unstimulated and stimulated TGFβ condition. (**B**) (left panel) Generation of a375m melanoma CRISPR/Cas9 *MEN1* KO. (Right panel) P21 expression upon short-term TGFβ stimulation in WM278 CRISPR/Cas9 scrambled or *MEN1* KOs (sg10 and sg14). Gene expression was measured by Western blotting. (**C**) Regulation of cell-cycle progression in tumor tissues from WM278 CRISPR/Cas9 SCR (scrambled) or *MEN1* KO mice. Changes in protein levels were assessed through Western blotting. (**D**) (upper panel) Annexin V/PI dual staining was used to determine the percentages of live, apoptotic, and dead cells induced by TGFβ treatment. (bottom panel) Quantification table displaying the percentage of live, apoptotic, and dead cells. Cell-cycle experiments were performed in three biological replicates. (**E**) (upper panel) Regulation of Caspase 3 expression in tumor tissues from WM278 CRISPR/Cas9 SCR (scrambled) or *MEN1* KO. Changes in protein levels were assessed through Western blotting. (bottom panel) Histogram showing the quantification by densitometry for each band relative to β-tubulin loading control. *, *p* < 0.05. **, *p* < 0.01.

TGFβ exerts its tumor-suppressive effects through regulation of the cell cycle, apoptosis, autophagy, and cell immortalization [30]. To gain further insights into the involvement of *MEN1* in the cell cycle, we performed flow cytometry analysis of propidium iodide-stained scrambled and *MEN1* KO WM278 cells treated or not treated with picomolar concentrations of TGFβ. The efficiency of all the KOs was tested by Western blotting (Figure 3A left panel). As shown in Figure 3A (right panel), in control cells (SCR), TGFβ significantly increased the number of cells in the G1 phase of the cell cycle, with concomitant decreases in the number of cells in the S and G2/M phases. However, in *MEN1* KO cells, the TGFβ-mediated increase in cells in G1 was reduced, while no TGFβ-mediated decrease in cells in the S phase was observed compared to that in cells in the scrambled group. Moreover, no significant differences were observed between the SCR and MEN1-KO conditions. Smad3 KO was used as a positive control and nearly completely blocked the effects of TGFβ. The effects of TGFβ on cell-cycle arrest are well characterized and involve the upregulation of several cyclin-dependent kinase inhibitors, such as p21 [31], with concomitant downregulation of c-myc [32]. Thus, we next assessed the effect of TGFβ on p21 expression in *MEN1* or SCR KO cells. While we found that TGFβ increased p21 gene expression in control (scr) cells, these effects were attenuated in the two *MEN1* KO cell lines (Figure 3B, right panel). The efficacy of *MEN1* KO was assessed by Western blotting (Figure 3B, left panel). To further assess the regulatory effect of *MEN1* on p21 and c-myc expression in a more clinically relevant system, we also examined p21 and c-myc expression levels in resected tumor samples from preclinical *MEN1* KO experiments (Figure 2C). Interestingly, as shown in Figure 3C, compared with control tumors, *MEN1* KO tumors exhibited significantly greater levels of c-myc and no detectable p21 (scr). These data are consistent with what was observed in vitro and collectively indicate that *MEN1* is required for a TGFβ-mediated increase in p21 expression, downregulation of c-myc expression, and cell-cycle arrest.

The tumor-suppressor effects of TGFβ in melanoma involve not only cell-cycle arrest but also induction of cell death by apoptosis [33]. To evaluate whether *MEN1* could also play a role in apoptosis, we assessed the effects of TGFβ on apoptosis using annexin V staining. As shown in Figure 3D, TGFβ treatment of WM278 melanoma cells led to an increase in apoptotic and dead cells. However, these effects were partially reversed in *MEN1* KO cells, similar to what was observed for Smad3 KO cells. By examining resected tumor samples from our in vivo transplantation experiments (Figure 2), we also found that, on average, the *MEN1* KO tumor samples exhibited lower caspase 3 levels than did the control tumors (Figure 3E). Taken together, these results strongly suggest that *MEN1* acts downstream of the TGFβ signaling pathway to regulate cell-cycle arrest and apoptosis in melanoma.

### 3.4. Identification of MEN1 Mutations in Melanoma Patients and Loss of TGFβ Responses

**Figure 4:**
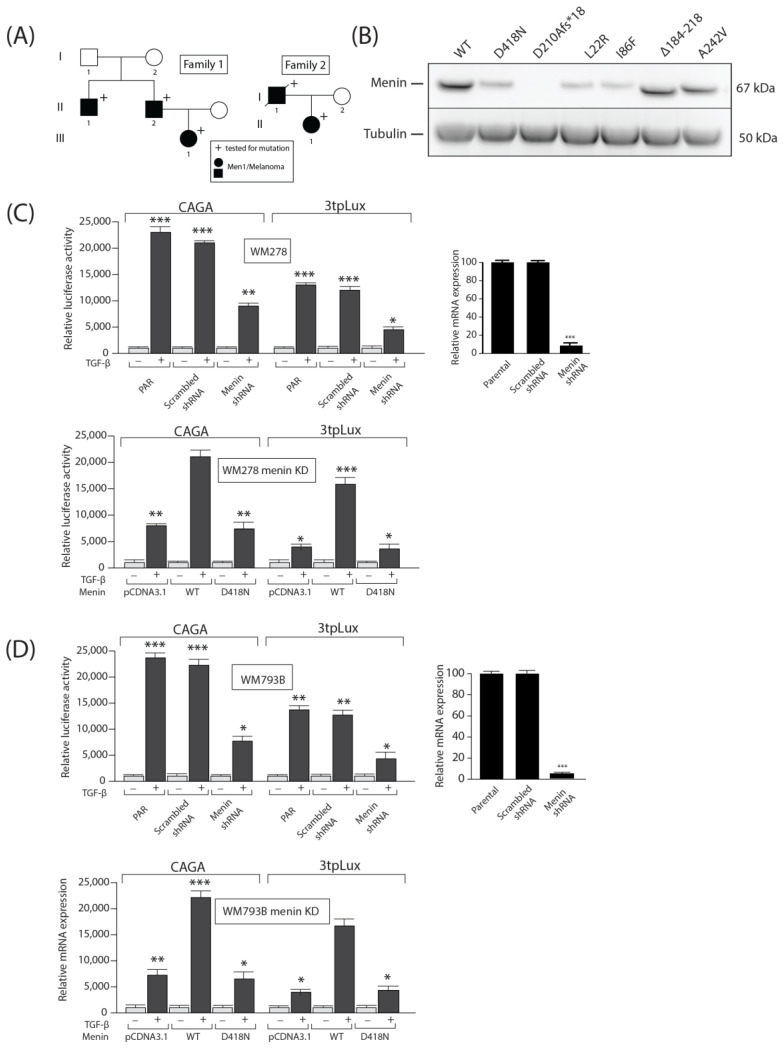
Expression of Menin mutants in HEK293 cells and generation of stable *MEN1* knockout melanoma cell lines. (**A**). Pedigrees of two MEN1 kindreds. (**B**). Flag-tagged Menin WT and mutant constructs were transfected into HEK293 cells, and after 48 h, the cell lysates were subjected to Western blot analysis with anti-Flag and anti-β-tubulin antibodies. (**C**). Efficiency of Menin knockdown in WM278 parental and shRNA-infected cells. Menin protein and mRNA (left panel) expression was measured via Western blot analysis and qPCR. TGFβ responsive CAGA and PAI-1 (3tpLlux) gene promoter activity in WM278 parental cells (right top panel) and WM278 *MEN1* KD cells (right lower panel). The data are graphed as the arithmetic mean of relative luciferase units normalized to β-galactosidase activity. (**D**). Efficiency of Menin knockdown in WM793B parental and shRNA-infected cells. Menin protein and mRNA (left panel) expression was measured via Western blot analysis and qPCR. TGFβ responsive CAGA and PAI-1 gene promoter activity in WM793B parental cells (right top panel) and WM793B *MEN1* KD cells (right lower panel). The data are graphed as the arithmetic mean of relative luciferase units normalized to β-galactosidase activity. *, *p* < 0.05. **, *p* < 0.01. ***, *p* < 0.001.

To gain further clinical insights into the role and contribution of *MEN1* in melanoma development, we identified melanoma patients harboring *MEN1* mutations. Family 1 (Figure 4A, left panel): The proband, a 61-year-old male (individual II-2), was admitted for a follow-up of pathologically diagnosed parathyroid carcinoma showing capsular invasion and infiltration into the esophagus. The proband’s serum ionized calcium concentration (iCal, mmol/liter) was 1.48 (normal range, 1.12–1.31), and her PTH level was 286 pg/mL (normal range, 10–65). At surgery, the enlarged parathyroid gland was removed. The proband was heterozygous for the recurrent missense mutation D418N in germline *MEN1*. This patient also developed in situ (scapula) melanoma. Assessment of first-degree relatives revealed the presence of hyperparathyroidism (hypercalcemia and hypercalciuria with high levels of PTH) in the proband’s brother (individual II-1) and daughter (individual III-1), and both were also heterozygous for the D418N mutation and developed melanomas. The proband’s brother underwent surgery for melanoma and lipoma. Family 2 (Figure 4A, right panel): The proband (individual II-1), a 34-year-old female, presented with a serum ionized calcium concentration (iCal, mmol/liter) of 1.41 (normal range, 1.12–1.31) and a PTH level of 215 pg/mL (normal range, 10–65), consistent with hyperparathyroidism. She underwent surgery for removal of the parathyroid adenoma. The proband was shown to be heterozygous for a novel germline *MEN1* deletion mutation, causing a frameshift leading to a truncated Menin protein (c.628_631delACAG (p.D210Afs*18)). This change was not found in 100 *MEN1* gene alleles from 50 unrelated normal individuals. The proband also developed melanoma in situ (arm), and the father of the proband (individual I-1) died from melanoma.

To study the expression and activity of the patients with *MEN1* mutations, we reproduced these patients’ mutations (D418N, D210Afs*18) in wild-type (WT) *MEN1* cDNA using in vitro site-directed mutagenesis. We also reproduced other well-characterized *MEN1* mutations (L22R, I86F, ∆184-218, A242V) as controls. These L22R and I86F mutants were previously shown to be unstable and were used here as positive controls, while the stable mutants (∆184-218 and A242) were used as negative controls [34]. As shown in Figure 4B, while WT *MEN1* and the positive controls (∆184-218 and A242V) were strongly expressed when transiently transfected into HEK293 cells, missense mutants (D418N and L22R) were expressed at much lower levels, while the frameshift mutant D210AAfs*18 was not expressed. Previous studies have shown that missense *MEN1* mutants can be degraded via the ubiquitin–proteasome pathway [34,35,36]. Thus, our results suggest that the *MEN1* mutation characterized in family 1 (proband individual II-1) may lead to the production of unstable *MEN1* products, further leading to their rapid degradation and further loss of TGFβ transcriptional responses. For the D210Afs*18 mutant from family 2 (proband individual II-1), the frameshift mutation led to a truncated Menin product lacking more than 50% of the protein, including the nuclear localization signal sequence. As such, that product is predicted to be unstable and rapidly degraded [37] and, as a result, cannot be overexpressed (Figure 4A).

We began addressing this by first reducing *MEN1* WT expression levels in WM278 melanoma cells using a shRNA-mediated knockdown (KD) strategy. As shown in Figure 4C, the efficiency of lentiviral infection and *MEN1* shRNA knockdown was confirmed at both mRNA and protein levels using qPCR and Western blotting, respectively. Moreover, as shown in Figure 4C, right top panel), decreasing *MEN1* expression significantly reduced the TGFβ transcriptional response to 2 different luciferase reporter constructs (CAGA and 3TPLux). To determine whether the mutant *MEN1* sequence could rescue the KD phenotype, WT and the missense mutant D418N were transiently transfected into WM278 KD cells. As shown in Figure 4B (right panel, bottom), while overexpression of WT *MEN1* restored TGFβ-induced luciferase activity in cells transfected with both promoter constructs, overexpression of the missense mutant D418N did not, presumably due to instability and rapid degradation. Similar results were obtained when *MEN1* was knocked down in WM793B cells (Figure 4D). These results indicate that the *MEN1* D418N mutation is functionally inactive in relaying TGFβ transcriptional responses.

### 3.5. The Expression and Activity of MEN1 Missense Mutants Can Be Partially Rescued by a Proteasome Inhibitor

**Figure 5:**
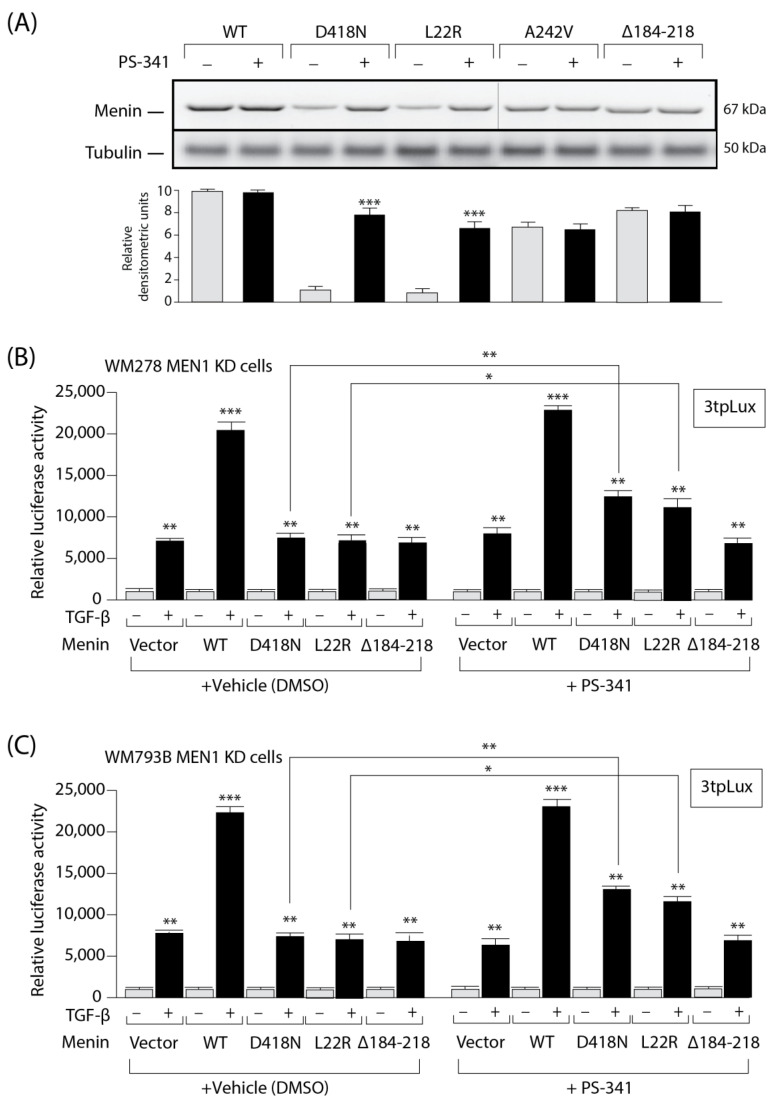
The expression and activity of Menin missense mutants can be rescued by the proteasome inhibitor PS-341. (**A**). Western blot analysis of HEK293T cells transfected with Flag-tagged WT or mutant Menin and treated (black) or not treated (gray) with PS-341 (Velcade). (**B**,**C**). TGFβ–responsive PAI-1 (3tpLux) gene promoter activity in WM278 and WM793B *MEN1* KD cells treated with either vehicle or PS341. The data are graphed as the arithmetic mean of relative luciferase units normalized to β-galactosidase activity. *, *p* < 0.05. **, *p* < 0.01. ***, *p* < 0.001.

Having shown that the *MEN1* D418N mutation failed to restore TGFβ signaling, we next investigated whether blocking its degradation could restore TGFβ responses and tumor suppression. The proteasome inhibitor PS-341 (Velcade, Bortezomib) is in clinical use for relapsed multiple myeloma and exhibits favorable selectivity toward tumors compared with normal cells [38]. As shown in Figure 5A, blocking the proteasome with the PS-341 inhibitor (for 4 h at 90 nM) partially restored the expression of the Menin missense mutant D418N and the L22R mutant, which were used here as a positive control. As expected, PS-341 had no effect on the WT or stable Menin mutants (A242V and ∆184-218).

To determine whether blocking the proteasome could offer therapeutic value for patients harboring *MEN1* mutations, we examined whether the expression of the unstable *MEN1* mutants and the TGFβ transcriptional response could be rescued using a proteasome inhibitor. For this purpose, we used the melanoma cell lines engineered above (Figure 5B,C; cells depleted of endogenous *MEN1* or overexpressing WT or mutant *MEN1*). KD cells were then transfected with the 3TPLux luciferase reporter before being stimulated with TGFβ. As shown in Figure 5, in vehicle (DMSO)-treated WM278 *MEN1* KD (5B) and WM793B *MEN1* KD (5C) melanoma cells, the TGFβ transcriptional response was enhanced in cells overexpressing WT Menin relative to that in cells harboring the empty vector, whereas no difference was observed in cells overexpressing *MEN1* mutants, confirming that the mutants failed to transmit the TGFβ responses. Interestingly, when WM278 *MEN1* KD and WM793B *MEN1* KD cells were treated with PS-341 for 6 hrs, the WT and the unstable *MEN1* mutants (D418N and L22R) were able to partially restore TGFβ responses, consistent with the partial restoration of *MEN1* mutant expression (Figure 5A). As expected, the negative control (a stable mutant [∆184-218]) had no effect. Thus, blocking the proteasome degradation pathway with a specific chemical inhibitor can restore both *MEN1* expression and the TGFβ response.

### 3.6. The Expression and Activity of MEN1 Missense Mutants Can Be Rescued by Inhibition of the Ubiquitin Ligase CHIP

**Figure 6:**
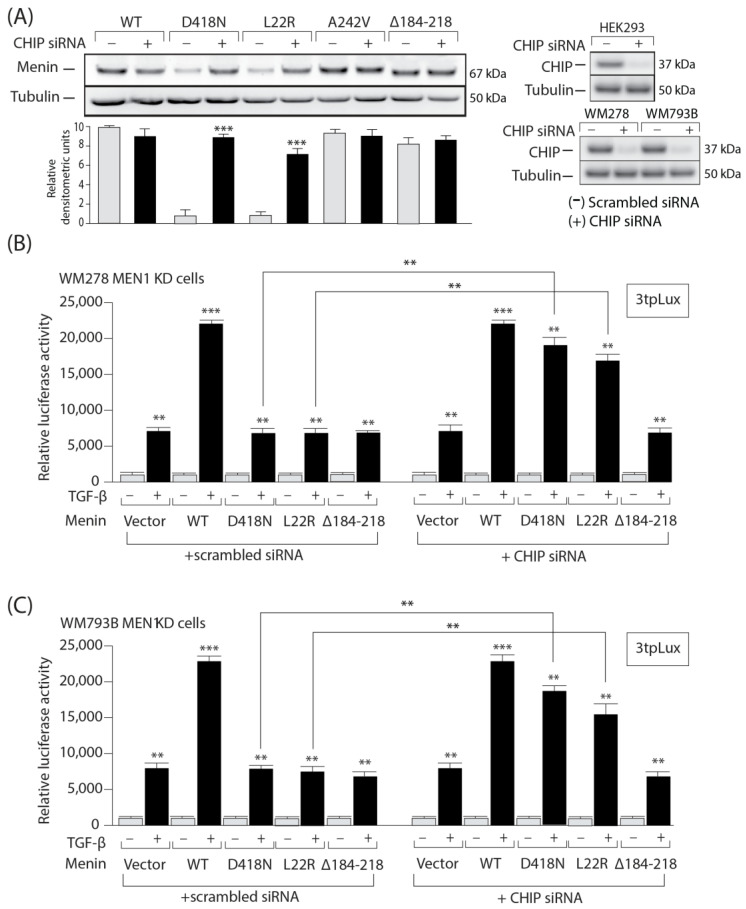
The expression and activity of Menin missense mutants can be rescued by RNAi targeting the ubiquitin ligase CHIP. (**A**). Western blot analysis of HEK293T cells transfected with Flag-tagged WT or mutant Menin and treated with scrambled (gray) or specific CHIP siRNA (black) (left panel). Western blot analysis of ChIP in HEK293, WM278 and WM793 cells treated with scrambled or specific CHIP siRNA (right panel). (**B**,**C**) TGFβ responsive PAI-1 (3tpLux) gene promoter activity in WM278 *MEN1* KD and WM793B *MEN1* KD cells transfected with scrambled siRNA or specific CHIP siRNA. The data are graphed as the arithmetic mean of relative luciferase units normalized to β-galactosidase activity. **, *p* < 0.01. ***, *p* < 0.001.

In parallel, to block the proteasome degradation pathway more specifically, we knocked down the expression of the C-terminal Hsp70 binding protein (CHIP) in melanoma cells. CHIP acts as a co-chaperone that can interact with the molecular chaperones Hsp70 and Hsp90, further leading to an imbalance of the folding-refolding machinery toward the degradation pathway [39]. Interestingly, blocking the proteasome through silencing CHIP gene expression with a specific siRNA completely restored the expression of the two *MEN1* mutants (DN418 and L22R) but had no effect on the WT or stable *MEN1* mutants (Figure 6A, left panel). The efficiency of the CHIP siRNA KD was verified by Western blotting (Figure 6A, right panel).

We next examined whether the missense mutation of *MEN1* and the resulting TGFβ transcriptional response could be reversed by silencing CHIP expression. WM278 *MEN1* KD and WM793B *MEN1* KD cells were co-transfected with a scrambled (control) or CHIP-specific siRNA and the 3TPLux luciferase reporter before being stimulated with TGFβ. As shown in Figure 6B,C, only WT *MEN1* could induce TGFβ-mediated luciferase activity in control WM278 *MEN1* KD and WM793B *MEN1* KD melanoma cells, consistent with the results observed with the proteasome inhibitor. However, both the WT and the missense *MEN1* mutants (D418N and L22R) were able to almost completely restore the TGFβ response, while the negative control (∆184-218) had no effect.

### 3.7. PS-341 Restores the Ability of Menin Missense Mutants to Mediate TGF-b Upregulation of the CDKI p15 and p21 Gene Promoters

**Figure 7:**
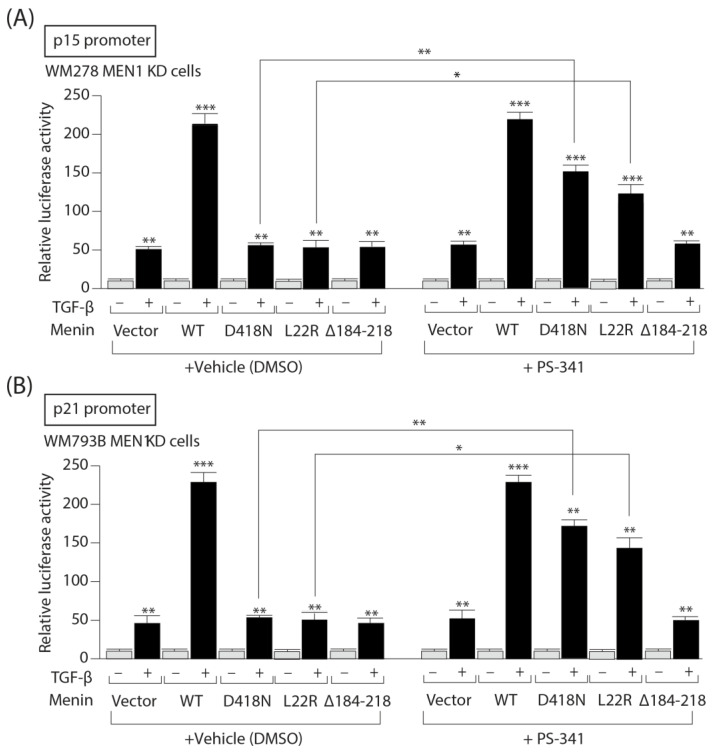
PS-341 restores the ability of Menin missense mutants to mediate TGF-β up-regulation of the p15 and p21 CDKI gene promoters. TGF-β responsive (**A**) p15 and (**B**) p21 gene promoter activity in WM278 *MEN1* KD cells treated with either vehicle or PS-341. The data are graphed as the arithmetic mean of relative luciferase units normalized to β-galactosidase activity. *, *p* < 0.05. **, *p* < 0.01. ***, *p* < 0.001.

Since TGF-b exerts, in part, its cytostatic actions by up-regulating the expression of CDKI genes such as p15 and p21, we examined whether treatment of Menin missense mutants with PS-341 would restore their ability to induce p15 and p21 gene promoter activities. As shown in Figure 7, in vehicle treated WM278 *MEN1* KD cell, although TGF-b stimulation of luciferase activity was enhanced by Menin WT relative to vector for both promoters p15 (Figure 7A) and p21 (Figure 7B), no enhancement occurred with Menin missense mutants. However, upon treatment with PS-341, the luciferase activities of both promoters were partially restored by Menin missense mutants (D418N and L22R). The negative control (D184-218) had no effect.

Taken together, these results indicate that the loss of TGFβ tumor-suppressive responses in patients harboring *MEN1* mutations leading to *MEN1* degradation could be circumvented by blocking the proteasome degradation pathway, thereby offering new therapeutic opportunities for patients with melanoma.

## 4. Discussion

This study highlights *MEN1* as a potent tumor-suppressor pathway that efficiently blocks tumorigenesis in cutaneous melanoma. Identification of specific point mutations in *MEN1* family members affected by melanoma also revealed increased *MEN1* gene product degradation, leading to a loss of TGFβ signaling. Using pharmacological inhibitors and RNA interference strategies, we showed that we could efficiently restore both *MEN1* gene expression and TGFβ signaling in melanoma cells. Our findings indicate that the use of currently FDA-approved drugs against proteasomal degradation and/or tailor-made therapies mimicking the TGFβ/Smad3/*MEN1* signaling pathway would be highly beneficial for melanoma patients, as these drugs would efficiently prevent initial tumor formation/progression and further hinder the spread of metastatic tumors to secondary organs.

Previous work highlighted *MEN1* as a downstream TGFβ signaling component that regulates the growth and proliferation of pituitary adenoma cells and osteoblasts [19,40,41]. Moreover, we showed that Menin is required for activin-mediated inhibition of PRL expression in pituitary cells [42]. A screening of secreted biomarkers during macrophage/melanoma interactions revealed activin A as a highly secreted factor, but not TGFβ1-2-3. This finding highlights the role of activin A in the oncogenic activation of monocytes and macrophages in the melanoma tumor-microenvironment and the upregulation of tumor-associated genes. The differential role of activin A in melanoma is underscored with higher expression being correlated with pro-tumorigenic effect in the early phase of skin tumorigenesis [43] and worse outcome in melanoma patients [44]. The present study expands on these findings, highlighting *MEN1* as a potent tumor suppressor downstream of TGFβ in nonendocrine tumors, such as melanoma. The results from our in vivo preclinical models clearly indicate that *MEN1* knockout leads to increased primary melanoma tumor growth. Thus, *MEN1* appears to function as a potent regulator of tumorigenesis in multiple endocrine and non-endocrine tissues, further revealing the broad range of biological processes regulated by *MEN1*. While TGFβ was found to play a dual role in promoting metastasis in breast cancer [44,45,46,47,48], TGFβ signaling has been shown to have antimetastatic effects on uveal melanoma [49], retinal Müller glia [50] and cutaneous melanoma. Thus, new therapeutic strategies aimed at activating the TGFβ/*MEN1* signaling pathway could prove useful for melanoma patients at different stages of the disease, including primary tumor formation.

Interestingly, multiple potential melanoma tumor suppressors are localized on chromosome 11q, which includes the *MEN1* region (located on chromosome 11q13) [51]. These results could suggest a possible association between *MEN1* and melanoma. To confirm this, our study highlights Menin as a potential tumor suppressor in melanoma. Furthermore, we report here two families with melanoma in which at least two first-degree family members tested positive for *MEN1* mutations. Interestingly, in addition to exhibiting typical *MEN1* endocrine tumors, these patients also developed melanoma, suggesting that *MEN1* is a strong candidate gene for familial malignant melanoma (for families in which 2 or more first-degree relatives, such as a parent, sibling, and/or child, exhibit skin cancer) [24,52]. The clinical implication is that patients should be tested for potential *MEN1* gene mutations whenever 2 or more family members have developed melanoma. Moreover, our study suggested that all patients testing positive for *MEN1* mutations are at risk of melanoma; because this may be a deadly disease, these patients should be monitored for melanoma. Still, a pan-cancer study reported that several mutations disabling MEN1 are somatic and that only a small fraction of germline mutation have the potential to be pathogenic and drive oncogenesis [53].

Characterization of the *MEN1* gene in two families bearing melanoma highlighted specific *MEN1* mutations, leading to loss of expression or increased degradation of the *MEN1* gene product, further leading to a loss of TGFβ signaling. Interestingly, twenty percent of *MEN1* cases involve Menin missense and small deletion or insertion mutations. The results from this study and work from others revealed that most of these mutants are expressed at markedly reduced levels relative to wild-type Menin. We found that blocking the proteasome degradation pathway with a specific proteasome inhibitor (PS-341, Velcade, or bortezomib) or with an RNA interference strategy aimed at silencing the expression of the molecular cochaperone CHIP efficiently restored *MEN1* expression and the TGFβ transcriptional response. Thus, specific gene silencing has the potential to provide additional therapies to those currently available for the treatment of melanoma. The use of small chemical inhibitors also appears to be very promising for melanoma treatment. The proteasome inhibitor PS-341 (Velcade, bortezomib) used in this study is already in clinical use for relapsed multiple myeloma [38,54]. Although proteasome inhibitors have multiple effects on apoptosis and cell proliferation, the present study provides proof of principle that future exploration of their use in treating subsets of *MEN1* patients is warranted.

## Figures and Tables

**Table 1:** Characteristics of cell lines.

Cell Line	Cell Type	Origin	Sex of Human Donor	Age	Mutations and Characteristics
WM278	Melanoma	Primary	Female	62	BRAF (V600E); PTEN (Deletion)
WM793B	Melanoma	Primary	Male	37	BRAF (V600E); CDK4 (K22Q); PTEN (Deletion)
BLM	Melanoma	Derived from lung metastases in nude mice injected with BRO parent cell line	Male	34	Nras (Q61R)
WM1232	Melanoma	Metastatic	Female	N/A	BRAF (V600E); PTEN (Deletion)
DAUV	Melanoma	Primary	N/A	N/A	BRAF (V600E)
SKMEL-28	Melanoma	Primary	Male	53	BRAF (V600E); CDK4 (R24C); EGFR (P753S); PTEN (T167A); TP53 (L145R); Tert (Promoter)
a375m	Melanoma	Isolated from a tumor from a nude mice injected with the parent a375 cell line	Female	54	BRAF(V600E); CDKN2A (Deletion); Tert (Promoter)
HEK293	Kidney	Embryo	Female	Fetus	

**Table 2:** Primer sequences for qPCR assay.

Gene		Sequence
MEN1	Forward	5′-GGAAGACGACGAGGAGATCTACA-3′
MEN1	Reverse	5′-CAGTAGTTCAGAGGCCTTTGCGCT-3′
GAPDH	Forward	5′-GCCTCAAGATCATCAGCAATGCCT-3′
GAPDH	Reverse	5′-TGTGGTCATGAGTCCTTCCACGAT-3′

**Table 3:** gRNA sequences for molecular cloning.

Gene		Sequence
MENsg1	Forward	5′-CACCGCACCTGCTGCGATTCTACGA-3′
MENsg1	Reverse	5′-AAACTCGTAGAATCGCAGCAGGTGC-3′
MEN2sg2	Forward	5′-CACCGACGTCGTCGATGGAGCGCAG-3′
MEN2sg2	Reverse	5′-AAACCTGCGCTCCATCGACGACGTC-3′
SCRsg1	Forward	5′-CACCGACGGAGGCTAAGCGTCGCAA-3′
SCRsg2	Reverse	5′-AAACTTGCGACGCTTAGCCTCCGTC-3′
SMAD2sg1	Forward	5′-CACCGTCCCACTGATCTATCGTATT-3′
SMAD2sg1	Reverse	5′-AAACAATACGATAGATCAGTGGGAC-3′
SMAD2sg2	Forward	5′-CACCGTGGCGGCGTGAATGGCAAGA-3′
SMAD2sg2	Reverse	5‘-AAACTCTTGCCATTCACGCCGCCAC-3′
SMAD3sg1	Forward	5′-CACCGCCCGATCGTGAAGCGCCTGC-3′
SMAD3sg1	Reverse	5′-AAACGCAGGCGCTTCACGATCGGGC-3′
SMAD3sg2	Forward	5′-CACCGTTCACGATCGGGGGAGTGAA-3′
SMAD3sg2	Reverse	5′-AAACTTCACTCCCCCGATCGTGAAC-3′
SMAD4sg1	Forward	5′-CACCGAACTCTGTACAAAGACCGCG-3′
SMAD4sg1	Reverse	5′-AAACCGCGGTCTTTGTACAGAGTTC-3′

## Data Availability

Uncropped versions of the Western blots are available as original data files. The other datasets generated during and/or analyzed during the current study are available from the corresponding authors upon reasonable request.
